# Mutations in Complement Regulatory Proteins Predispose to Preeclampsia: A Genetic Analysis of the PROMISSE Cohort

**DOI:** 10.1371/journal.pmed.1001013

**Published:** 2011-03-22

**Authors:** Jane E. Salmon, Cara Heuser, Michael Triebwasser, M. Kathryn Liszewski, David Kavanagh, Lubka Roumenina, D. Ware Branch, Tim Goodship, Veronique Fremeaux-Bacchi, John P. Atkinson

**Affiliations:** 1 Autoimmunity and Inflammation Program, Hospital for Special Surgery, Cornell Weill Medical College, New York, New York, United States of America; 2 Department of Obstetrics and Gynecology, University of Utah, Salt Lake City, Utah, United States of America; 3 Department of Medicine/Division of Rheumatology, Washington University School of Medicine, St. Louis, Missouri, United States of America; 4 Institute of Human Genetics, Newcastle University, Newcastle upon Tyne, United Kingdom; 5 Assistance Publique-Hopitaux de Paris, Hôpital Européen Georges-Pompidou, Service d'Immunologie Biologique, Paris, France; University of Queensland, Australia

## Abstract

**Background:**

Pregnancy in women with systemic lupus erythematosus (SLE) or antiphospholipid antibodies (APL Ab)—autoimmune conditions characterized by complement-mediated injury—is associated with increased risk of preeclampsia and miscarriage. Our previous studies in mice indicate that complement activation targeted to the placenta drives angiogenic imbalance and placental insufficiency.

**Methods and Findings:**

We use PROMISSE, a prospective study of 250 pregnant patients with SLE and/or APL Ab, to test the hypothesis in humans that impaired capacity to limit complement activation predisposes to preeclampsia. We sequenced genes encoding three complement regulatory proteins—membrane cofactor protein (MCP), complement factor I (CFI), and complement factor H (CFH)—in 40 patients who had preeclampsia and found heterozygous mutations in seven (18%). Five of these patients had risk variants in MCP or CFI that were previously identified in atypical hemolytic uremic syndrome, a disease characterized by endothelial damage. One had a novel mutation in MCP that impairs regulation of C4b. These findings constitute, to our knowledge, the first genetic defects associated with preeclampsia in SLE and/or APL Ab. We confirmed the association of hypomorphic variants of MCP and CFI in a cohort of non-autoimmune preeclampsia patients in which five of 59 were heterozygous for mutations.

**Conclusion:**

The presence of risk variants in complement regulatory proteins in patients with SLE and/or APL Ab who develop preeclampsia, as well as in preeclampsia patients lacking autoimmune disease, links complement activation to disease pathogenesis and suggests new targets for treatment of this important public health problem.

**Study Registration:**

ClinicalTrials.gov NCT00198068

*Please see later in the article for the Editors' Summary*

## Abstract 2

Jane Salmon and colleagues studied 250 pregnant patients with SLE and/or antiphospholipid antibodies and found an association of risk variants in complement regulatory proteins in patients who developed preeclampsia, as well as in preeclampsia patients lacking autoimmune disease.

## Editors' Summary

**Background:**

Most pregnancies culminate in the birth of a healthy baby but, sadly, about a quarter of women lose their babies during pregnancy. A common pregnancy-related medical problem that threatens the life of both baby and mother is preeclampsia. Mild and severe preeclampsia affects up to 10% and 1%–2% of pregnancies, respectively. Preeclampsia occurs because of a problem with the function of the placenta, the organ that transfers nutrients and oxygen from mother to baby and removes waste products from the baby. Although preeclampsia begins early in pregnancy, it is diagnosed by the onset of high blood pressure (hypertension) and the appearance of protein in the urine (proteinuria) after 20 weeks of pregnancy. Other warning signs include headaches and swelling of the hands and face. The only cure for preeclampsia is delivery, and labor is usually induced early to prevent eclampsia (seizures), stroke, liver and kidney failure, and breathing and blood vessel problems developing in the mother. Although delivery before 37 weeks of pregnancy is not generally recommended, in cases of preeclampsia it may be too dangerous for both the baby and the mother to allow the pregnancy to continue. Unfortunately when severe preeclampsia occurs in the second trimester, babies weighing only 500 grams may be delivered and they may not survive.

**Why Was This Study Done?:**

Because the exact cause of preeclampsia is unknown, it is difficult to develop treatments for the condition or to find ways to prevent it. Many experts think that immune system problems—in particular, perturbations in complement activation—may be involved in preeclampsia. The complement system is a set of blood proteins that attacks invading bacteria and viruses. The activation of complement proteins is usually tightly regulated (overactivation of the complement system causes tissue damage) and, because preeclampsia may run in families, one hypothesis is that mutations (genetic changes) in complement regulatory proteins might predispose women to preeclampsia. In this study, the researchers test this hypothesis by sequencing genes encoding complement regulatory proteins in pregnant women with the autoimmune diseases systemic lupus erythematosus (SLE) and/or antiphospholipid antibodies (APL Ab) who developed preeclampsia. In autoimmune diseases, the immune system attacks healthy human cells instead of harmful invaders. Both SLE and APL Ab are characterized by complement-mediated tissue injury and are associated with an increased risk of preeclampsia and miscarriage.

**What Did the Researchers Do and Find?:**

Two hundred fifty women with SLE and/or APL Ab were enrolled into the PROMISSE study (a multi-center observational study to identify predictors of pregnancy outcome in women with SLE and/or APL Ab) when they were 12 weeks pregnant and followed through pregnancy. Thirty patients developed preeclampsia during the study and ten more had had preeclampsia during a previous pregnancy. The researchers sequenced the genes for complement regulatory proteins: membrane cofactor protein (MCP), factor I, and factor H in these 40 patients. Seven women (18%) had mutations in one copy of one of these genes (there are two copies of most genes in human cells). Five mutations were alterations in MCP or factor I that are gene variants that increase the risk of hemolytic uremic syndrome, a disease characterized by blood vessel damage. The sixth mutation was a new MCP mutation that impaired MCP's ability to regulate complement component C4b. The final mutation was a factor H mutation that did not have any obvious functional effect. No mutations in complement regulatory proteins were found in 34 matched participants in PROMISSE without preeclampsia but, among a group of non-autoimmune women who developed preeclampsia during pregnancy, 10% had mutations in MCP or factor I.

**What Do These Findings Mean?:**

These findings identify MCP and factor I mutations as genetic defects associated with preeclampsia in pregnant women with SLE and/or APL Ab. Importantly, they also reveal an association between similar mutations and preeclampsia in women without any underlying autoimmune disease. Taken together with evidence from previous animal experiments, these findings suggest that dysregulation of complement activation is involved in the development of preeclampsia. Although further studies are needed to confirm and extend these findings, these results suggest that proteins involved in the regulation of complement activation could be new targets for the treatment of preeclampsia and raise the possibility that tests could be developed to identify women at risk of developing preeclampsia.

**Additional Information:**

Please access these Web sites via the online version of this summary at http://dx.doi.org/10.1371/journal.pmed.1001013.

- Tommy's, a UK charity that funds scientific research into the causes and prevention of miscarriage, premature birth, and stillbirth, has information on preeclampsia
- The March of Dimes Foundation, a nonprofit organization for pregnancy and baby health, has information on preeclampsia
- The UK National Health Services Choices website also has information about preeclampsia
- Wikipedia has pages on the complement system, on autoimmune disease, and on preeclampsia (note that Wikipedia is a free online encyclopedia that anyone can edit; available in several languages)
- More information on the PROMISSE study is available

## Introduction

Preeclampsia complicates 4%–5% of all pregnancies worldwide, causing significant maternal and neonatal mortality, and claims the lives of >60,000 mothers each year in developing countries [1]. Although typically diagnosed with the onset of hypertension and proteinuria after 20 weeks' gestation, the syndrome begins earlier in pregnancy with abnormal placental development. The specific “anatomical” defect, the failure of uterine spiral arteries to remodel into dilated, flaccid vessels, leads to underperfusion of the intervillous space and placental hypoxia. The clinical manifestations of preeclampsia represent the maternal response to an excess of antiangiogenic factors released by the hypoperfused placenta. These factors include vasculopathic factors such as soluble fms-like tyrosine kinase 1 (sFlt-1), a potent vascular endothelial growth factor (VEGF) antagonist, and soluble endoglin, an inhibitor of TGF-β signaling [2],[3]. Disease manifestations range from mild blood pressure elevations to severe hypertension, the HELLP syndrome (hemolysis, elevated liver enzymes, and low platelets), or eclampsia (seizures). The molecular basis for placental dysregulation is unknown and treatment is limited. Higher rates of preeclampsia in sisters, daughters, and mothers of affected women suggests a genetic contribution to risk for disease, and evidence for paternal components of predisposition supports the hypothesis that the genotype of the fetus contributes to the overall risk of preeclampsia [4].

**Figure 1:**
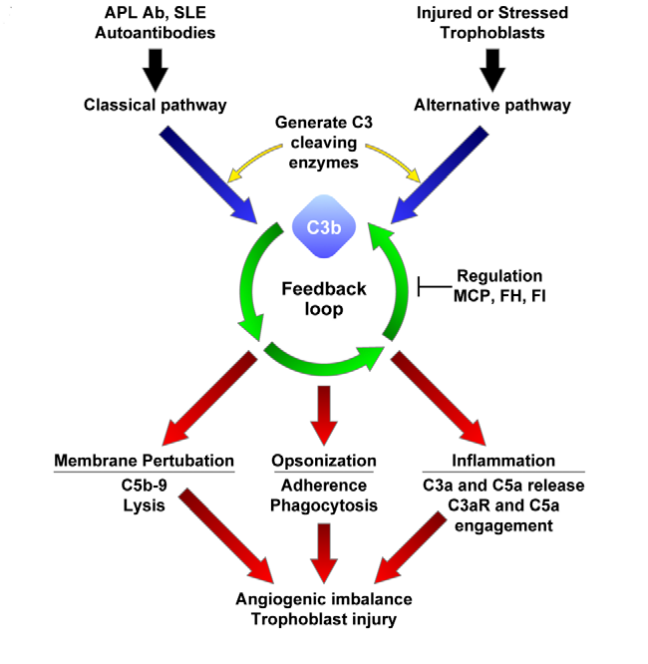
Complement activation in preeclampsia. Black and blue arrows indicate triggering of complement cascades by autoantibodies or damaged self-tissue; green arrows, deposition of C3b on a target sets in motion the powerful amplification loop of the alternative pathway; red arrows, effector activities of complement are generated by C3b deposition and C3a release and the downstream mediators C5b-9 and C5a. Regulation of feedback loop of alternative pathway on the placental trophoblasts (or endothelial cells) occurs through limited proteolytic cleavage of C3b to generate iC3b. This reaction is carried out by a serine protease, factor I (FI), along with membrane cofactor protein (MCP) or CFH (FH). Because SLE and APS are characterized by autoantibodies that trigger the classical pathway, defective regulation of C4b (a component of the classical pathway C3 convertase) by MCP is also likely to influence the severity of tissue injury and risk for preeclampsia. If regulators such as MCP, CFI (FI), or CFH (FH) are dysfunctional, excessive complement activation occurs. This may result in placental damage, thrombobosis and release of antiangiogenic factors, culminating in preeclampsia.

Immunologic maladaption has been proposed as a pathogenic mechanism for preeclampsia, but remains an unproven theory. We have previously established a link between the complement system and angiogenic factor imbalance associated with placental dysfunction (Figure 1). Studies in mouse models of pregnancy indicate that complement activation targeted to the placenta drives angiogenic imbalance, placental insufficiency, and endothelial injury [5]. In our experimental model, complement C5a-C5a receptor interactions trigger release of sFlt-1. This antiangiogenic factor is associated with hypertension, proteinuria, and glomerular endotheliosis in rodents [2],[3],[6] and is elevated in the circulation of pregnant women destined for preeclampsia. Further, in mice with abnormal pregnancies, the alternative complement pathway amplifies fetoplacental injury [5]. Also, uncontrolled complement activation due to deficiency of a membrane complement regulator analogous to human MCP is embryonic lethal because of maternal complement-triggered damage, and pregnancies are rescued in mice with reduced alternative pathway function [7]. Elevated levels of the alternative pathway complement activation fragment Bb in the first 20 weeks of pregnancy was recently found to be independently associated with preeclampsia later in pregnancy [8] and underscores the importance of complement, particularly the alternative pathway, in this syndrome.

Pregnancy in women with systemic lupus erythematosus (SLE) or the antiphospholipid syndrome (APS), two autoimmune diseases characterized by complement-mediated injury, is associated with an increased risk of preeclampsia, placental insufficiency, fetal growth restriction, and miscarriage [9]. In these patients, autoantibodies targeted to the placenta initiate local complement activation which, if unabated, leads to abnormal placental development (Figure 1). Indeed, complement activation products have been demonstrated in deciduas, chorionic villi, and vessel walls in the placentas of patients with APL Ab and in patients with preeclampsia [10],[11]. Moreover, evidence of alternative pathway activation has been found in the circulation of pregnant SLE patients. In normal pregnancies, excessive complement activation is prevented by complement regulatory proteins that are highly expressed on trophoblast membranes (membrane cofactor protein [MCP (CD46)], decay accelerating factor [DAF (CD55)], and CD59) as well as circulating complement regulatory proteins (complement factor H [CFH], C4b binding protein, and complement factor I [CFI]) (Figure 1) [11]. We hypothesized that impaired capacity to limit complement activation predisposes pregnant women with SLE or APL Ab to preeclampsia.

Loss-of-function mutations in CFH, MCP, and CFI or gain-of-function mutations in complement components factor B and C3 lead to undesirable complement activation in patients with atypical hemolytic uremic syndrome (aHUS), a microangiopathy characterized by microvascular endothelial activation, cell injury, and thrombosis [12],[13],[14],[15]. Complement dysregulation (specifically, enhanced function of the alternative pathway) contributes to the pathology of aHUS, a disease associated with glomerular endothelial cell injury and microthrombi, not unlike the pathologic findings in preeclampsia, APS, and SLE. Complement mutations in aHUS demonstrate incomplete penetrance (∼50%), indicating that additional genetic and environmental factors are required to manifest disease. Pregnancy, SLE, and/or APL Ab may provide these additional factors. The aims of this study are to translate our findings in mouse models to patients and determine whether variations in complement regulatory proteins leading to impaired function are associated with preeclampsia in patients with SLE and/or APL Ab and whether such variants are also present in women who develop preeclampsia in the absence of autoimmune disease.

## Methods

### Participants

The first group of patients studied were enrolled in The PROMISSE Study (**P**redictors of p**R**egnancy **O**utcome: bio**M**arkers **I**n antiphospholipid antibody **S**yndrome and **S**ystemic lupus **E**rythematosus), a prospective multi-center observational study to identify predictors of pregnancy outcome initiated in 2003. Patients with SLE (defined as ≥4 ACR criteria) and/or APL Ab (defined as at least one of following documented twice between 6 wk and 5 y apart: ACL [IgG or IgM ≥40 units], lupus anticoagulant, or anti-β2GPI [IgG or IgM ≥40 units]) and disease controls were recruited by 12 wk gestation. At screening, patients with renal disease (proteinuria >1,000 mg/24 h, RBC casts, or serum creatinine >1.2 mg/dl), taking prednisone >20 mg/d, diabetes mellitus (Type I and Type II antedating pregnancy), hypertension (blood pressure ≥140/90 mmHg), or multiple gestations were excluded. Detailed medical and obstetrical information and serial blood specimens were obtained monthly during the course of pregnancy. Preeclampsia was defined as new onset of elevated systolic blood pressure (≥140 mmHg) and/or elevated diastolic blood pressure (≥90 mmHg) after 20 wk gestation on two occasions at least 4 h apart and proteinuria of 300 mg or greater in a 24 h urine specimen or ≥1+ on dipstick at least 4 h apart in the absence of pyelonephritis or hematuria. Severe preeclampsia and HELLP are defined by standard criteria [16]. In women with proteinuria present in early gestation, preeclampsia was defined as an increase in proteinuria to double their baseline level. For patients with a history of preeclampsia in previous pregnancies, medical charts were reviewed to document that patients met the above criteria above.

Of 250 enrolled patients with SLE and/or APL Ab who completed their pregnancies before 31 May 2008, 30 patients developed preeclampsia during the study and 10 additional patients had documented preeclampsia in a previous pregnancy (Table 1). Three of the 30 patients with preeclampsia during the PROMISSE Study pregnancy had documented preeclampsia in a previous pregnancy. None of the disease-free controls developed preeclampsia. The racial/ethnic distribution of the 40 patients with preeclampsia was 67% white, 17% black, 7% Asian, 5% Hispanic, and 4% other.

**Table 1:** Preeclampsia in autoimmune PROMISSE patients.

Clinical Characteristics	No. Enrolled	No. with Preeclampsia in PROMISSE	No. with Preeclampsia in Previous Pregnancy^a^	Total Patients with Preeclampsia^b^	% Patients with Preeclampsia
SLE only	149	19	5	23	15%
APL Ab only	59	6	5	10	17%
SLE with APL Ab	32	5	3	7	22%
**Total**	**250**	**30**	**13**	**40**	**17%**

a The PROMISSE Study pregnancy was the first pregnancy in 40% of the SLE patients (with and without APL Ab) and in 9% for APL Ab patients without SLE. The APL Ab patients differed from SLE patients with regard to multiparity because pregnancy loss is a criterion for testing for APL Ab.

b Three patients had preeclampsia in the PROMISSE Study and also had preeclampsia in previous pregnancies.

The Institutional Review Board at each of the PROMISSE Study sites (Hospital for Special Surgery, NY, NY, US; NYU School of Medicine, NY, NY, US; Johns Hopkins University, Department of Rheumatology, Johns Hopkins Medical Institutions, Baltimore, MD, US; Mount Sinai Hospital, Toronto, Canada; University of Utah, Intermountain Health Care, Salt Lake City, UT, US; Oklahoma Medical Research Foundation, University of Oklahoma Health Sciences Center, Oklahoma City, OK, US) gave approval for human participation for patients. Written informed consent that included the use of material for genetic research was obtained from all PROMISSE participants who contributed samples toward this study. All study samples were deidentified.

The second group of patients studied was from the Universal Samples Database at University of Utah, which stores maternal blood and clinical data from women who present to labor and delivery. Charts were reviewed to confirm the diagnosis of severe preeclampsia and HELLP defined according to established criteria, and 59 patients meeting criteria and with available DNA were identified [16]. The racial/ethnic distribution of patients with severe preeclampsia and/or HELLP was 74% white (non-Hispanic), 25% Hispanic, and 1% other. Control pregnancies defined as uncomplicated term deliveries in women with no more than one pregnancy loss and at least one live birth were identified in the Universal Samples Database at University of Utah. The Institutional Review Board at University of Utah, Salt Lake City, UT, gave approval for human participation for the participants whose samples are stored at the Universal Samples Database at University of Utah. Samples from 143 ethnically matched controls were available.

The allele frequency of MCP A304V and CFI IVS12+5 G>T variants in white individuals were determined using DNA from The UK Blood Services collection of Common Controls (UKBS collection) which forms part of the Wellcome Trust Case Control Consortium (WTCCC) (http://www.wtccc.org.uk) [17],[18].

### Genetic and Statistical Analyses

Genomic DNA was extracted from peripheral blood leukocytes, and coding sequence and intronic flanking regions of MCP, CFH, and CFI were screened as described [19],[20]. Mutations considered for our analysis were either missense or nonsense coding mutations and mutations in conserved positions of splice sites and promoters. To further restrict our study to mutations that are likely deleterious, we ignored mutations present in dbSNP (http://www.ncbi.nlm.nih.gov/projects/SNP/), except for those previously observed in aHUS. This left novel variants and known aHUS alleles with the described properties. Mutations that occur in less than 5% of the population are termed low-frequency variants, and those in <0.5% are rare variants [21].

The rare variants could not be tested for Hardy-Weinberg equilibrium (HWE) because they do not appear in controls. The A304V variant was tested and found to be in HWE in 40 PROMISSE and 59 Utah cases (*p* = 1.0) and in the WTCCC controls (*p* = 0.15). The 34 PROMISSE controls could not be assessed for HWE because no alleles were present, and the 143 Utah controls were found not to be in HWE, largely because of a single homozygote that was sampled (*p* = 0.02).

To test the hypothesis that A304V is associated with preeclampsia, we combined cases and controls from the PROMISSE and Utah studies. These two populations were combined because the frequencies of A304V were similar, and Fisher's exact test was used to test for enrichment of a deleterious, dominant allele.

### Expression and Functional Assessment of MCP

Substitution mutagenesis was performed using the QuikChange site-directed mutagenesis kit (Stratagene). The MCP template was the MCP isoform BC1 (GenBank accession no. X59405) cloned into the EcoRI site of plasmid pCDNA3.1+ (Stratagene). Transient transfections were performed with Fugene-6 (Roche Molecular Biochemicals) into Chinese hamster ovary (CHO) K1 cells. After cell lysis, MCP was quantified in an ELISA as described [22]. Briefly, MCP mAb GB24 was coated at 2.5 µg/ml on microtiter wells followed by blocking in PBS containing 4% BSA and 0.05% Tween 20. Cell lysates and standards were incubated in wells followed by reaction with a rabbit anti-MCP antiserum, horseradish- peroxidase–labeled donkey anti-rabbit IgG (Jackson ImmunoResearch), and ImmunoPure TMB Substrate kit (Pierce).

Ligand binding and cofactor assays for C4b and C3b have been described [22]. Briefly, an ELISA format was used for characterizing ligand binding. C3b or C4b (Complement Technologies) was coated onto wells of a microtiter plate. Next, dilutions of MCP wild-type or mutant CHO lysates were added. Cofactor assays employed biotinylated ligands in low- or physiologic salt buffer (i.e., 10 mM Tris [pH 7.4], with 25 or 150 mM NaCl), CFI (100 ng), and cell lysates (25 pg of wild-type or mutant MCP). After 20 and 90 min, cleavage fragments were analyzed by a 10% size fractionation on reducing SDS/PAGE followed by transfer, Western blotting, and densitometric scanning [22].

## Results

### Patients with Preeclampsia

The PROMISSE Study is a prospective multi-center observational study in which patients with SLE and/or APL Ab were enrolled at 12 wk gestation and followed through pregnancy. Patients with renal insufficiency, diabetes mellitus (antedating pregnancy), hypertension, or multiple gestations (risk factors predisposing to preeclampsia) were excluded. Between 2003 and 2008, 250 patients with SLE and/or APL Ab completed their pregnancies, 40 of whom developed preeclampsia during the study or had preeclampsia in a previous pregnancy (Table 1). All patients with preeclampsia were screened for mutations in the genes encoding the complement regulatory proteins MCP, CFI, and CFH. Mutations that occur in less than 5% of the population (low-frequency alleles) are termed uncommon variants [21].

We identified heterozygous mutations in complement system pathway genes in seven of 40 patients with preeclampsia (18%)—four patients with mutations in MCP, two with CFI mutations, and one with a CFH mutation. Complement C3 levels were normal at enrollment in these patients. We did not find any mutations in 34 patients without preeclampsia (15 matched for disease [SLE and/or APL Ab], age, and ethnicity, and 19 healthy control patients matched for age and ethnicity) (7/40 versus 0/34, *p*<0.01; also see results of mutation screening described for each gene in the sections to follow). We reject the null hypothesis of equal or fewer rare, deleterious alleles in controls at the 0.02 level (*p* = 0.0105 for the one-tailed test). This approach represents a variation of existing collapsing methods for the analysis of low-frequency variants [23]. The frequency of these mutations and variants in other healthy populations is described below. The clinical characteristics of patients with mutations are presented in Table 2.

**Table 2:** Clinical characteristics of autoimmune patients with complement regulatory protein mutations.

Mutation/Variant	Autoimmune Disease	GA at Delivery (wk)	Other Pregnancies
MCP/A304V	SLE	35.4^a^	3 early losses, 1 live birth
MCP/A304V	SLE, APL Ab	29.0^b^	None
MCP/A304V	APL Ab	37.3	2 live births, 1 with preeclampsia
MCP/K32N	SLE	25.0	2 early losses, 1 live birth
CFI/IVS12+5 G>T	APL Ab	29.0^c^	1 live birth, preterm at 24 weeks
CFI/IVS12+5 G>T	SLE	37.5	1 miscarriage; 1 elective termination
CFH/S40A	SLE	37.4	None

Patients were white, except the patient with MCP K32N, who was black. Characteristics of preeclampsia [16].

a Severe preeclampsia with visual disturbance.

b Preeclampsia with oligohydramnios and intrauterine growth restriction (<5th percentile).

c Severe preeclampsia, HELLP syndrome.

GA, gestational age.

To replicate the relationship of complement system variants with preeclampsia and to determine whether mutations in complement regulatory proteins are present in non-autoimmune patients with severe preeclampsia and/or HELLP, we screened 59 patients and 143 ethnically matched healthy controls with normal pregnancies from the Universal Samples Database at the University of Utah for mutations in MCP, CFI, and CFH. We identified heterozygous mutations in the genes of five patients—four in MCP and one in CFI. No significant sequence variants were detected in CFH. The clinical characteristics of the patients from this cohort are presented in Table 3. Three individuals among the healthy controls had the A304V variant (5/59 versus 3/143, *p*<0.05). Details of the mutations and variants and their distribution are described below.

**Table 3:** Clinical characteristics of non-autoimmune preeclampsia patients with complement regulatory protein mutations.

Mutation/Variant	Maternal Age (y)	Preeclampsia	GA at Delivery (wk)	Fetal Weight in Grams (Percentile weight for GA)
MCP/A304V	24	Severe	33	2,102 (27)
MCP/A304V	23	Severe^a^	34	2,807 (48)
MCP/A304V	41	HELLP^b^	22	Neonatal demise
MCP/A304V	33	Severe^c^	33	2,013 (22)
CFI/I398L	24	Severe^d^	37	2,940 (35); 2,145 (2)

All patients were white.

a Previous pregnancy preeclampsia delivered at 36 wk.

b Pregnancy complicated by sphenoid sinus thrombosis; thrombophilia evaluation negative; four normal previous pregnancies; history of idiopathic thrombocytopenia purpura; mother with SLE.

c Oligohydramnios.

d Twins.

GA, gestational age.

### Mutations and Variants of MCP

MCP is a widely expressed transmembrane protein that binds C3b and C4b deposited on cell surfaces and serves as a cofactor for their cleavage by CFI. The resulting attached fragments, iC3b and C4d, are not capable of forming convertases. By cleaving C3b, MCP is a key regulator on host cells of the amplification loop of the alternative pathway. Four patients with preeclampsia (10%) had mutations in MCP. Three patients were heterozygous for MCP A304V, a low-frequency hypomorphic variant previously shown to be deficient in control of alternative pathway activation on the cell surface [14]. The allele occurred at a frequency of 3.7% in the PROMISSE Study patients with preeclampsia. All three patients with A304V were white, and one had SLE only, one APL Ab only, and one both SLE and APL Ab. Two patients had severe preeclampsia and two patients reported prior pregnancy complications, including preeclampsia (Table 2). This variant has been identified in four of 238 patients from the French multi-center aHUS cohort and in two of 181 healthy French controls, and in none of 120 white participants from another study [12]. Because the frequency of MCP A304V was not reported in publically available databases, including HapMap, we genotyped a large population of whites. In 1,529 individuals from the WTCCC, the allele frequency was 2.1% (61 heterozygotes and two homozygotes). Of note, no pregnancy histories or other medical information was available for these individuals.

We confirmed the association of MCP A304V with preeclampsia in a second cohort identified from the Universal Samples Database at the University of Utah. In 59 non-autoimmune patients with severe preeclampsia and/or HELLP, we identified four individuals (6.8% of patients) heterozygous for MCP A304V (allele frequency 3.4%). The details of their pregnancies are presented on Table 3. In 144 ethnically and geographically matched controls with uncomplicated pregnancies, two individuals were heterozygous and one homozygous for MCP A304V (allele frequency 1.4%), none of whom had histories of thrombotic microangiopathy or preeclampsia. The frequency of MCP A304V was consistent with our findings in the WTCCC. Combining our autoimmune and non-autoimmune cohorts with preeclampsia (*n* = 99), we identified seven individuals heterozygous for MCP A304. In both groups separately and when combined (99 cases versus 178 controls), A304V confers risk of preeclampsia. The variant acts in a dominant fashion with an odds ratio of 4.4 (95% confidence interval 1.1–17.6; *p* = 0.027).

We have previously studied in detail the complement inhibitory profile of A304V employing permanent CHO cell lines expressing an equal copy number of wild-type or A304V. While there was no detectable difference in ELISA type assays of solubilized protein, in situ the A304V was ∼50% as effective at controlling C3b deposition by the alternative pathway [14]. In addition, a 25%–50% reduction in levels of wild-type complement regulatory proteins expressed by CHO cells impairs regulation of the alternative pathway, showing that haploinsufficiency has functional consequences [14],[24].

**Figure 2:**
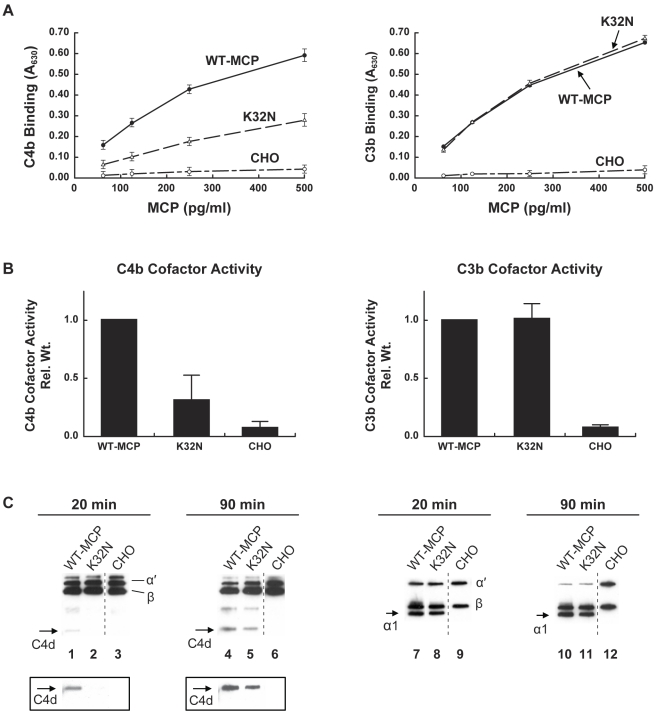
C3b and C4b binding and cofactor activity of K32N compared to wild type MCP. (A) C4b and C3b binding. CHO cell lysates from transient transfections were incubated with C4b- or C3b-coated wells and binding was assessed using a polyclonal antibody to MCP in an ELISA format. CHO, a mock transfected control. Data represent the mean ± standard deviation at each concentration from three independent experiments. (B) Cofactor activity for CFI-mediated cleavage of C4b and C3b. Cell lysates containing 25 pg of MCP (wild-type or K32N) were incubated with biotinylated C4b or C3b in the presence of purified human CFI in 25 mM NaCl and cleavage was assessed by Western blot. C4b and C3b peptide chains and cleavage fragments are identified. For C4b cofactor activity, C4d fragment generated by cleavage of C4b after 90 min was quantified by densitometric scanning, compared with quantity of β chain, and normalized to wild type. For C3b cofactor activity, α1 fragment generated by cleavage was compared to β chain by densitometry, and normalized to wild type. Data represent the mean ± standard deviation of three independent experiments. (C) Representative Western blots of MCP activity for CFI-mediated cleavage of C4b and C3b. Left panel: MCP K32N has deficient C4b cofactor activity at 20 and 90 min. The C4d fragment generated by cleavage of the α′ chain is not visible at 20 min and is diminished relative to wild type at 90 min. The boxed inserts represent 5-fold longer exposure of the blot. The CHO negative control was a nonadjacent lane (broken line). Right panel: MCP K32N has similar C3b cofactor activity to wild type. Equal amounts of α1 fragments are generated after 20 and 90 min. The CHO negative control was a nonadjacent lane (broken line). In two additional experiments, similar results were obtained in cofactor assays at 150 mM NaCl concentration (unpublished data).

The potential role of complement dysregulation in preeclampsia is underscored by our discovery of a novel mutation in exon 2 of the gene encoding MCP (K32N) in a patient from the PROMISSE Study who had SLE. Her pregnancy ended with preeclampsia and fetal death at 25 wk. She had a history of two early pregnancy losses. MCP K32N is a missense mutation that alters a charged solvent-exposed amino acid in complement control protein repeat (CCP) 1 [25]. To examine its functional consequences, the K32N mutant construct was transiently transfected in CHO cells. The mutant protein was quantified in cellular lysates and binding to human C4b and C3b was assessed (Figure 2A). While binding to C3b was similar to wild-type MCP, the mutant showed an approximately 50% decrease in C4b binding (*p*<0.001) compared to wild-type MCP. Given the lower binding to C4b, we suspected that the K32N mutant form of MCP would have a decreased capacity to serve as a cofactor for CFI-mediated cleavage of C4b. To examine this possibility, we performed cofactor assays comparing the ability of wild-type MCP and rare mutant K32N to serve as cofactors for cleavage of C3b and C4b by monitoring loss of the α′ chain and generation of cleavage fragments (C4d in the case of C4b and α1 in the case of C3b) (Figure 2B) [24]. As predicted by the binding studies, cofactor activity for C3b was similar for the K32N mutant and wild-type MCP, whereas the K32N mutant showed decreased CFI-mediated cleavage activity for C4b. At 20 min in the C4b cofactor activity assay, no C4d was detected in the K32N lane, but an easily discernable band in wild-type MCP. We performed densitometric scanning of the C4d after 90 min and found that cleavage of C4b to C4d in wild-type was four times greater than in the K32N mutant (Figure 2C). Our results define the functional defect in K32N and identify the initial disease-related mutation in MCP that results exclusively in impaired ability to bind C4b and mediate C4b cofactor activity. Because SLE is characterized by autoantibodies that trigger the classical pathway, defective regulation of C4b is likely to influence the severity of tissue injury and risk for preeclampsia (Figure 1). Indeed, the presence of C4 degradation products in placentas from patients with APL Ab and in kidneys from lupus patients underscores the importance of C4 in disease pathogenesis [10],[26].

### Mutation in CFI

CFI, a plasma glycoprotein, is a serine protease that cleaves the α′ chain of C3b and C4b in the presence of a cofactor protein, such as CFH or MCP. Inactivation of C3b and C4b prevents the formation of C3 and C5 convertases and thereby down-modulates the classical, lectin, and alternative pathways. We found identical heterozygous mutations in the gene encoding CFI in two unrelated white women with preeclampsia in the PROMISSE Study (Table 2). Similar to three of the four patients with MCP variants, those with CFI mutations had a history of previous pregnancy complications, specifically preterm deliveries at 24 and 28 wk, respectively. The mutation identified in CFI, an IVS12+5 G>T change, has been reported in patients with aHUS and was shown to affect the donor site of intron 12 reducing the splice score from 93 to 86 [27] but did not alter CFI serum levels [12]. The CFI IVS12+5 G>T variant has been identified in six of 325 French control individuals (0.9% allele frequency) and in eight of 238 patients from the French multi-center aHUS cohort (1.7% allele frequency) (alone in four patients and in association with a second mutation in four cases; V. Fremeaux-Bacchi, unpublished results). The frequency of this variant is not reported in publically available databases. Our findings suggest that CFI IVS12+5 G>T is a low- frequency variant that may be enriched in aHUS. Its clinical impact cannot be assessed in the control patients because pregnancy histories and other medical information were not available for these individuals.

The two patients presented here and other patients with the IVS12+5 G>T variant have had normal CFI levels (66 and 63 mg/l, nl 42-78 mg/l), but CFI levels within the normal range does not exclude alterations in local production [28],[29]. In the 12 Eutherian mammals that have a similar CFI gene architecture to human CFI (Ensemble release 52), conservation is perfect at the +5 intron position. The lack of variation at this position relative to the surrounding bases argues that a G at this base is functionally important.

We screened CFI in 57 non-autoimmune patients with severe preeclampsia and/or HELLP in our second cohort from the Universal Samples Database at University of Utah. In one patient, we identified a heterozygous nonsynonymous sequence variant in exon 11 (c.1246A>G, p.Ile398Leu). We did not find this change in 117 white British controls. Bienaime et al. and Sellier-Leclerc et al. have previously demonstrated this variant in three aHUS patients (two heterozygous, one homozygous) but not in 100 controls [29],[30]. CFI was undetectable in the individual with the homozygous change [29]. Recombinant expression of the I398L mutant confirmed nearly complete absence of production compared to wild-type CFI, which was associated with altered subcellular localization detected by immunofluorescence and altered endoglycosidase H digestion [29]. We identified two other nonsynonymous changes in this cohort: c. 1217G>A, p.Arg388His [19],[28] and c.1322A>G, p.Lys423Arg [rs41278047]. Both changes have previously been described in control populations and are classed as SNPs in our study. Identification of CFI I398L mutation, a complete loss of function leading to haploinsufficiency, supports a role for defects in complement regulation by CFI in the pathogenesis of preeclampsia. This association also suggests that enrichment of IVS12+5G>T variants in patients with preeclampsia and aHUS [12] is functionally important, although its mechanism is as yet undefined.

### Mutations in Factor H

The single factor H mutation (S40A) in the PROMISSE cohort was extensively investigated and not shown to have a functional defect (normal secretion in a transfection system, normal plasma levels, and normal to increased C3b binding and cofactor activity) (unpublished data). No factor H mutations were found in the Utah cohort.

## Discussion

MCP and CFI mutations are, to our knowledge, the first genetic defects to be associated with preeclampsia in pregnant patients with SLE and/or APL Ab. Our hypothesis—that mutations affecting function (missense, nonsense, and conserved splice site and promoter mutations) in negative regulators of the amplification loop of complement would be enriched in preeclampsia as compared with controls—is supported by our finding that such mutations in these genes carries an odds ratio of 8.2 (95% CI 1–69) in the PROMISSE study cohort. We replicated the association of mutations in genes encoding complement inhibitors with preeclampsia in a second cohort of patients with severe preeclampsia and/or HELLP, but without underlying autoimmune disease. Taken together, our results suggest that dysregulation of complement activation can be a risk for preeclampsia.

Mutations in *MCP* and *CFI* appear to predispose to the development of disease, and as observed in aHUS, mutations are heterozygous and penetrance is incomplete [12]. In both preeclampsia and aHUS, a second hit that initiates the complement cascade or endothelial injury is required to manifest disease. In PROMISSE Study patients, autoantibodies may lower the threshold for preeclampsia, resulting in clinically apparent disease. These autoantibodies trigger the classical pathway of complement locally and systemically. Complement activation is then amplified through the feedback loop of the alternative pathway, leading to further injury of the placenta and stimulation of endothelial cells to express a prothrombotic, proadhesive phenotype (Figure 1). In patients with obesity or diabetes, conditions that predispose to preeclampsia and are characterized by systemic inflammation, the inflammatory process may sensitize vascular endothelium to injury. In aHUS, infection or pregnancy may trigger complement and precipitate disease [12],[31]. Indeed, in four patients with impaired complement regulatory protein function, pregnancy precipitated thrombotic microangiopathy in the form of atypical HELLP with severe renal involvement [32],[33]. Our findings in patients in the PROMISSE Study, taken together with case series of aHUS and HELLP patients, argue that pregnancy per se provokes or amplifies thrombotic microangiopathy in those already at risk due to inadequate control of the complement system.

In sequencing only three complement regulatory genes, we found heterozygous mutations in 10%–20% of PROMISSE patients with preeclampsia, which likely underestimates the importance of genetic defects in complement regulation, because we analyzed only the maternal genes, although trophoblasts are of fetal origin and express both maternal and paternal genes. Also, we have yet to analyze CFB and C3, which account for 10%–15% of the mutations in aHUS [13],[15]. Our findings, in this prospective, longitudinal study of patients at risk for preeclampsia (in contrast to a biased sample from a case series), demonstrate that mutations or uncommon variants in complement regulatory proteins are not rare in those who develop disease and support the possibility that they increase disease risk. Confirmation of the presence of hypomorphic uncommon variants and mutations in a cohort of non-autoimmune patients with severe preeclampsia (the Utah cohort) provides further evidence for the importance of complement activation in disease pathogenesis.

Based on findings in our patients and prior experiments in mouse models, we propose the following mechanism for the genesis of preeclampsia. Defective regulation of the complement system allows for the excessive complement activation that leads to placental damage, abnormal placental development, generalized endothelial activation, and the release of antiangiogenic factors toxic to the fenestrated endothelium of glomeruli, the choroid plexus, and liver sinusoids—a sequence of events that culminates in clinical preeclampsia (Figure 1). Future studies of the PROMISSE cohort will determine whether elevations of split products generated by activation of the alternative or classical complement pathway predict preeclampsia in patients with APL Ab and/or SLE, and the information in the current report will allow us to refine these analyses and compare those with and without mutations leading to excessive complement activation. Our findings underscore the important role of complement activation in preeclampsia, define mutations and likely mechanisms for increased risk in patients with SLE and/or APL Ab, and suggest new targets for treatment of this important public health problem that, thus far, has defied reliable prediction and satisfactory intervention.

## Abbreviations

aHUS: atypical hemolytic uremic syndrome
APL Ab: antiphospholipid antibody
APS: antiphospholipid syndrome
C5aR: C5a receptor
CCP: complement control protein
CFH: complement factor H
CFI: complement factor I
CHO: Chinese hamster ovary
HWE: Hardy-Weinberg equilibrium
MCP (or CD46): membrane cofactor protein
PROMISSE: **P**redictors of p**R**egnancy **O**utcome: bio**M**arkers **I**n antiphospholipid antibody **S**yndrome and **S**ystemic lupus **E**rythematosus)
sFlt-1: soluble fms-like tyrosine kinase 1
SLE: systemic lupus erythematosus
sVEGFR-1: soluble VEGF receptor-1
VEGF: vascular endothelial growth factor
WTCCC: Wellcome Trust Case Control Consortium

## References

[1] Ilekis JV, Reddy UM, Roberts JM (2007). Preeclampsia—a pressing problem: an executive summary of a National Institute of Child Health and Human Development workshop.. Reprod Sci.

[2] Maynard S, Epstein FH, Karumanchi SA (2008). Preeclampsia and angiogenic imbalance.. Annu Rev Med.

[3] Young BC, Levine RJ, Karumanchi SA (2010). Pathogenesis of preeclampsia.. Annu Rev Pathol.

[4] Esplin MS, Fausett MB, Fraser A, Kerber R, Mineau G (2001). Paternal and maternal components of the predisposition to preeclampsia.. N Engl J Med.

[5] Girardi G, Yarilin D, Thurman JM, Holers VM, Salmon JE (2006). Complement activation induces dysregulation of angiogenic factors and causes fetal rejection and growth restriction.. J Exp Med.

[6] Qing X, Redecha PB, Burmeister MA, Tomlinson S, D'Agati VD (2010). Targeted inhibition of complement activation prevents features of preeclampsia in mice.. Kidney Int 2010 Oct 13 [Epub ahead of print].

[7] Mao D, Wu X, Deppong C, Friend LD, Dolecki G (2003). Negligible role of antibodies and C5 in pregnancy loss associated exclusively with C3-dependent mechanisms through complement alternative pathway.. Immunity.

[8] Lynch AM, Gibbs RS, Murphy JR, Byers T, Neville MC (2008). Complement activation fragment Bb in early pregnancy and spontaneous preterm birth.. Am J Obstet Gynecol.

[9] Tincani A, Bazzani C, Zingarelli S, Lojacono A (2008). Lupus and the antiphospholipid syndrome in pregnancy and obstetrics: clinical characteristics, diagnosis, pathogenesis, and treatment.. Semin Thromb Hemost.

[10] Shamonki JM, Salmon JE, Hyjek E, Baergen RN (2007). Excessive complement activation is associated with placental injury in patients with antiphospholipid antibodies.. Am J Obstet Gynecol.

[11] Girardi G, Bulla R, Salmon JE, Tedesco F (2006). The complement system in the pathophysiology of pregnancy.. Mol Immunol.

[12] Caprioli J, Noris M, Brioschi S, Pianetti G, Castelletti F (2006). Genetics of HUS: the impact of MCP, CFH, and IF mutations on clinical presentation, response to treatment, and outcome.. Blood.

[13] Noris M, Remuzzi G (2009). Atypical hemolytic-uremic syndrome.. N Engl J Med.

[14] Fang CJ, Fremeaux-Bacchi V, Liszewski MK, Pianetti G, Noris M (2008). Membrane cofactor protein mutations in atypical hemolytic uremic syndrome (aHUS), fatal Stx-HUS, C3 glomerulonephritis, and the HELLP syndrome.. Blood.

[15] Kavanagh D, Richards A, Atkinson J (2008). Complement regulatory genes and hemolytic uremic syndromes.. Annu Rev Med.

[16] (2002). ACOG practice bulletin. Diagnosis and management of preeclampsia and eclampsia. Number 33, January 2002.. Obstet Gynecol.

[17] (2007). Genome-wide association study of 14,000 cases of seven common diseases and 3,000 shared controls.. Nature.

[18] Burton PR, Clayton DG, Cardon LR, Craddock N, Deloukas P (2007). Association scan of 14,500 nonsynonymous SNPs in four diseases identifies autoimmunity variants.. Nat Genet.

[19] Fremeaux-Bacchi V, Dragon-Durey MA, Blouin J, Vigneau C, Kuypers D (2004). Complement factor I: a susceptibility gene for atypical haemolytic uraemic syndrome.. J Med Genet.

[20] Fremeaux-Bacchi V, Moulton EA, Kavanagh D, Dragon-Durey MA, Blouin J (2006). Genetic and functional analyses of membrane cofactor protein (CD46) mutations in atypical hemolytic uremic syndrome.. J Am Soc Nephrol.

[21] Manolio TA, Collins FS, Cox NJ, Goldstein DB, Hindorff LA (2009). Finding the missing heritability of complex diseases.. Nature.

[22] Liszewski MK, Leung M, Cui W, Subramanian VB, Parkinson J (2000). Dissecting sites important for complement regulatory activity in membrane cofactor protein (MCP; CD46).. J Biol Chem.

[23] Li B, Leal SM (2008). Methods for detecting associations with rare variants for common diseases: application to analysis of sequence data.. Am J Hum Genet.

[24] Liszewski MK, Leung MK, Schraml B, Goodship TH, Atkinson JP (2007). Modeling how CD46 deficiency predisposes to atypical hemolytic uremic syndrome.. Mol Immunol.

[25] Casasnovas JM, Larvie M, Stehle T (1999). Crystal structure of two CD46 domains reveals an extended measles virus-binding surface.. Embo J.

[26] Cohen D, Koopmans M, Kremer Hovinga IC, Berger SP, Roos van Groningen M (2008). Potential for glomerular C4d as an indicator of thrombotic microangiopathy in lupus nephritis.. Arthritis Rheum.

[27] Rogozin IB, Milanesi L (1997). Analysis of donor splice sites in different eukaryotic organisms.. J Mol Evol.

[28] Kavanagh D, Richards A, Noris M, Hauhart R, Liszewski MK (2008). Characterization of mutations in complement factor I (CFI) associated with hemolytic uremic syndrome.. Mol Immunol.

[29] Bienaime F, Dragon-Durey MA, Regnier CH, Nilsson SC, Kwan WH (2010). Mutations in components of complement influence the outcome of Factor I-associated atypical hemolytic uremic syndrome.. Kidney Int.

[30] Sellier-Leclerc AL, Fremeaux-Bacchi V, Dragon-Durey MA, Macher MA, Niaudet P (2007). Differential impact of complement mutations on clinical characteristics in atypical hemolytic uremic syndrome.. J Am Soc Nephrol.

[31] Fakhouri F, Roumenina L, Provot F, Sallee M, Caillard S (2010). Pregnancy-associated hemolytic uremic syndrome revisited in the era of complement gene mutations.. J Am Soc Nephrol.

[32] Fakhouri F, Jablonski M, Lepercq J, Blouin J, Benachi A (2008). Factor H, membrane cofactor protein, and factor I mutations in patients with hemolysis, elevated liver enzymes, and low platelet count syndrome.. Blood.

[33] Fang CJ, Richards A, Liszewski MK, Kavanagh D, Atkinson JP (2008). Advances in understanding of pathogenesis of aHUS and HELLP.. Br J Haematol.

